# Reviewed article: Borger MASB, Zara ALSA, Tomich LGMM, Guilarde AO, Oliveira CP, Carvajal DLM, Pedrosa MMR, Costa PSS, Turchi MD. COVID-19 case fatality ratio and survival among hospitalized adults in Goiás, 2020: a cohort study. Epidemiol Serv Saude. 2025:34;20240053. 

**DOI:** 10.1590/S2237-96222025v34e20240053.a

**Published:** 2025-05-23

**Authors:** Tayanny Margarida Menezes Almeida Biase

**Affiliations:** Universidade Estadual de Campinas, School Pharmaceutical Science

**Table:** 

Rating	Excellent	Good	Average	Below average	Poor
Interest			✓		
Quality			✓		
Originality		✓			
Overall		✓			

**Table:** 

Questionnaire	Yes	No	Not applicable
Does the manuscript contain new and significant information to justify publication?	✓		
Does the Abstract (Summary) clearly and accurately describe the content of the article?		✓	
Is the problem significant and concisely stated?	✓		
Are the methods described comprehensively?		✓	
Are the interpretations and conclusions justified by the results?	✓		
Is adequate reference made to other work in the field?	✓		

## Manuscript Structure

Length of article is: Adequate

Number of tables is: Adequate

Number of figures is: Adequate

Please state any conflict(s) of interest that you have in relation to the review of this paper (state “none” if this is not applicable): None

## Comments

O trabalho descreve aspectos clínicos, estima a letalidade e fatores de risco para óbito em adultos hospitalizados com covid-19 em Goiás. É um trabalho relevante por avaliar uma região brasileira com poucos estudos relacionados ao tema. Os autores realizaram métodos estatísticos robustos e apresentaram diversos resultados que podem auxiliar melhorias na gestão de saúde de síndromes respiratórias.

1. Introdução

Padronização para citar referências. Antes ou após a pontuação, verificar normas da revista.

Padronizar o termo Covid-19, ora iniciando com maiúscula, ora minúscula.

Linha 41-16: reformular construção, sugiro iniciar falando que a letalidade por covid no Brasil foi X, já que os dois estudos referenciados foram realizadas no país.

Linha 53-55: jargões “contudo, embora”.

2. Métodos

Os métodos precisam ser descritos com mais detalhes para permitir a replicação do estudo por outros pesquisadores.

Linha 23: sugiro desmembrar o tópico de tipo de estudo, contexto e população. É importante esclarecer qual a população de Goiânia, quantos hospitais possui? Dentre outras informações relevantes para o contexto do estudo.

Linha 49: sigla SIVEPE-Gripe não descrita.

Sugiro seguir guia de redação conforme o check-list Strobe.

Minimização de vieses: foi empregado ajustes para fatores de confusão. As variáveis com um valor de p ≤ 0,10 na análise bivariada foram incluídas no modelo de riscos proporcionais de Cox para ajustar possíveis fatores de confusão.

Instrumento de coletada de dados validados: o texto não informa se houve validação ou treinamento da equipe para a coleta de dados. Os dados foram obtidos da notificação no SIVEPE-Gripe e pela revisão de prontuários clínicos e registros hospitalares.

Informação sobre perdas: a amostra foi de conveniência e não-probabilística e pode representar uma pequena parcela dos pacientes hospitalizados. Os autores citam essa limitação na discussão do estudo.

3. Resultados

Linha 49-52: adotar um padrão para citar os resultados. Se iniciou citando os valores encontrados, ex “651 indivíduos”, sugiro manter o padrão, ex “414 internados durante a pandemia” Linha 4-11: parágrafo dispensável. Trata-se da legenda das tabelas. Sugiro mencionar Tabela 1 ou 2 no momento em que os resultados referentes a cada uma forem chamados no texto.

Linha 48-53: padronizar tamanho da fonte.

4. Discussão

Linha 53-55: evitar jargões, está implícito que dados são embasados na literatura. Sugiro descrever brevemente os estudos que tiveram resultados semelhantes e fazer associação.

5. Figuras e tabelas

Baixa resolução da figuras 1 e 2.

